# Supplementary material for: Graphene Oxide Nanofiltration Membranes Containing Silver Nanoparticles: Tuning Separation Efficiency via Nanoparticle Size
Source: Nanomaterials (Basel). 2020 Mar 3;10(3):454. doi: 10.3390/nano10030454 (PMC7152991; doi:10.3390/nano10030454)
Supplement: Supplementary file 1 [file nanomaterials-10-00454-s001.pdf]

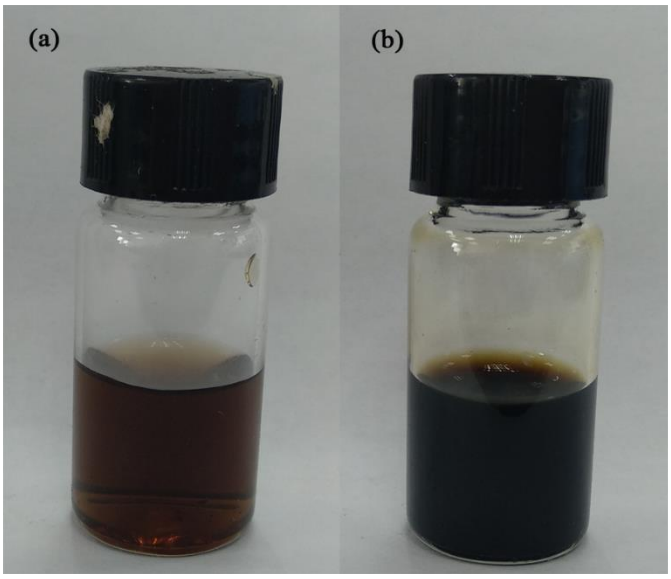

Fig. S1 (a) GO (b) GO/AgNPs composite solution.

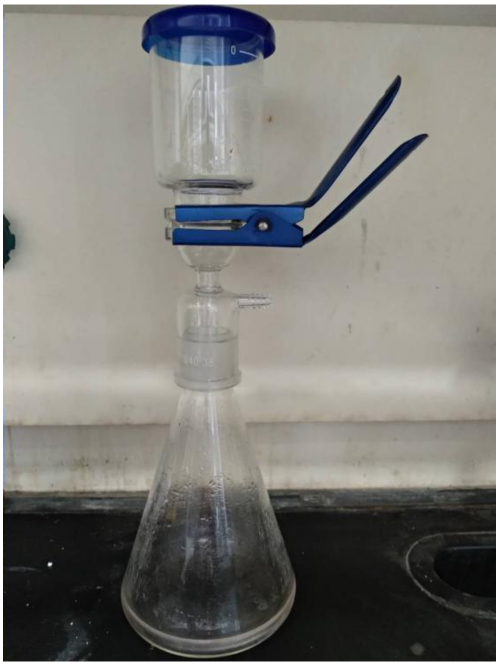

Fig. S2 The homemade dead-end filtration device for vacuum filtration.

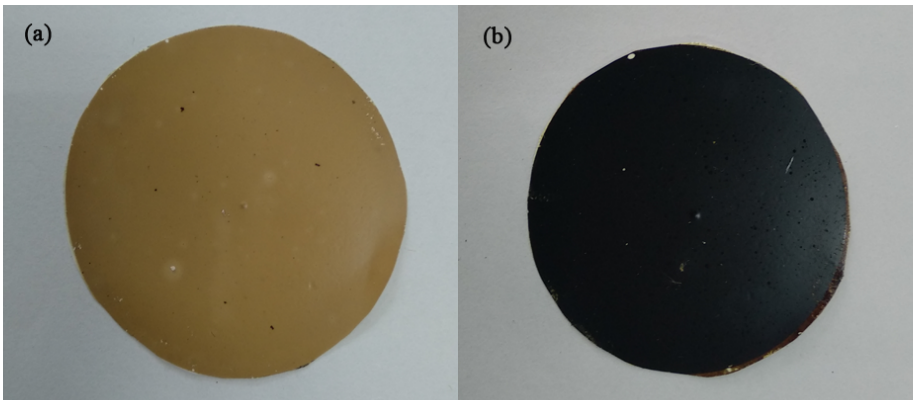

Fig. S3 (a) GO membrane (b) GO/AgNPs composite membrane by vacuum filtration.
